# Tumour‐targeting bacteria‐based cancer therapies for increased specificity and improved outcome

**DOI:** 10.1111/1751-7915.12787

**Published:** 2017-08-03

**Authors:** Sebastian Felgner, Vinay Pawar, Dino Kocijancic, Marc Erhardt, Siegfried Weiss

**Affiliations:** ^1^ Infection Biology of Salmonella Helmholtz Centre for Infection Research Braunschweig Germany; ^2^ Molecular Bacteriology Helmholtz Centre for Infection Research Braunschweig Germany; ^3^ Institute of Immunology Medical School Hannover Hannover Germany

## Abstract

‘You have cancer’ – a devastating diagnosis that still strikes patients hard. Despite substantial improvements of standard therapies over the years, there is still no general cure available. Here, we review the revival of an old concept – the use of bacteria as cancer therapeutics. Bacteria‐mediated tumor therapy has great potential to evolve into a powerful tool against malignant solid tumors.

‘You have cancer’ – a devastating diagnosis that still strikes patients hard. Despite substantial improvements of standard therapies over the years, there is still no general cure available. Thus, cancer represents an enormous burden on modern societies – socially as well as economically. According to recent statistics from the National Cancer Institute, every second human being will receive the diagnosis of cancer throughout his lifetime and every fourth will succumb to the disease (Howlader *et al*., 2014). For this year, the American Cancer Society predicts that approximately 600 000 Americans are expected to die of cancer – that is more than 1650 people a day. This only gives a glimpse of the suffering and emotional strain of the patient and his social environment. Besides, estimates of the direct medical costs for cancer in the United States were $87.8 billion in 2014. Even more striking is the prognosis. By 2030, 50% more cancer cases will be diagnosed and mortality due to this disease will increase by 60%. One major reason for this increasing threat is the demographic chance of the society. As cancer is essentially a disease of old age, with improved live expectancy, the chance to develop cancer is increasing accordingly. For such reasons, the introduction of novel efficacious therapies is a task of utmost importance for modern biomedical research. These efforts are well in agreement with the sustainable developments demanded by the WHO especially with the challenge: ensure healthy lives and promote well‐being for all at all ages.

Here, we review the revival of an old concept – the use of bacteria as cancer therapeutics. Bacteria‐mediated tumour therapy has great potential to evolve into a powerful tool against malignant solid tumours.

To date, early diagnosis and classical treatment options like surgery, radio‐ and chemotherapy represent the backbone of cancer therapy. Although such therapies have been continuously improved, they bear many disadvantages and risks (Crawford, 2013). For instance, not every tumour can be surgically removed and the unspecific activity of radio‐ and chemotherapies causes serious damage to healthy tissue. This renders such therapies suboptimal. The major disadvantage of the classical tumour therapies, however, remains the absence of a complete and sustainable cure, i.e. prevention of tumour relapse or inability to clear metastases and micrometastases. Therefore, in the past decades, research focus has shifted to more specific therapies that aim to fight cancer by, e.g., targeting molecular characteristics of the tumour or using the specificity of the immune system by employing antibodies or specific T cells (Weiner *et al*., 2010; Perica *et al*., 2015). Another example of an immunotherapeutic concept that is at the verge of general acceptance is the use of genetically modified bacteria against solid tumours. Such microorganisms exhibit natural tumour‐targeting as well as adjuvant abilities (Forbes, 2010).

Bacteria‐mediated tumour therapy (BMTT) is not a new concept. The roots of this therapy reach beyond introduction of radiotherapy for cancer treatment. Already at the end of the 19^th^ century, the US physician W. Coley explored the potential of bacteria for treatment of patients suffering from inoperable skin tumours (Hoption Cann *et al*., 2003). Coley was quite successful. However, it was difficult to control the bacterial infections at that time as antibiotics had not yet been discovered. In addition, many experts questioned Coley's approach as he was not able to explain the mode of action of this therapy. The idea to use bacterial pathogens to treat tumours was considered foolish and still is by many oncologists (McCarthy, 2006). In particular in the light of the fact that some bacteria such as *Helicobacter, Salmonella* Typhi or *Fusobacteria* can induce or support cancer (Ray, 2011; Kostic *et al*., 2013; Nagaraja and Eslick, 2014; Wang *et al*., 2014), the approach of BMTT was considered irrational. For a long time, it appeared to be impossible to find a balance between therapeutic benefit, safety and risks.

However, these concerns about BMTT did not discourage other members of the scientific community to renew research on this targeted therapy and first successes became apparent recently. These were primarily due to new tools of genetic engineering, as well as the improved understanding of host–pathogen interactions and tumour biology and resulted in different approaches to treat tumours using bacteria. An outstanding example is BCG (*Bacillus* Calmette‐Guerin), a highly selected strain of *Mycobacterium bovis* (Redelman‐Sidi *et al*., 2014). It was found to prevent relapses of superficial bladder cancer to a high degree and has been deployed in the clinics already since the late 1980s. Further promising candidates for BMTT include Gram‐positive bacteria like *Listeria monocytogenes* or *Clostridium novyi*‐NT and Gram‐negative bacteria like *Salmonella* Typhimurium and *Escherichia coli* (Paterson *et al*., 2010; Kubiak and Minton, 2015; Felgner *et al*., 2016a; Kocijancic *et al*., 2016). Although the concepts to apply the various bacteria for cancer therapy differ significantly, they have all been able to demonstrate antitumour properties in preclinical models and some even in clinical trials.

For instance*, Listeria* strains like ANZ‐100 or CRS‐207 are predominantly used as cancer vaccines that stimulate a T‐cell‐dependent anti‐tumour response. Recent clinical trials with CRS‐207 expressing mesothelin showed promising results in patients with pancreatic cancer (Maciag *et al*., 2009; Le *et al*., 2012).

Similarly, the use of *Clostridia* spores in BMTT was recently investigated in the clinic. It had been demonstrated that such spores can colonize human and canine tumours to induce anti‐tumour effects (Krick *et al*., 2012; Roberts *et al*., 2014). They were even able to breach the blood–brain barrier in a rat glioma multiforma model (Staedtke *et al*., 2015). Although the exact mechanisms of tumour colonization and anti‐tumour effects of *Clostridia* spores are poorly understood, they are taken as safe vectors as spores of these obligate anaerobic bacteria can only germinate in necrotic cores of tumours that lack oxygen (Barbé *et al*., 2006). However, this confinement to anoxia also represents a limitation because small tumours often lack necrotic regions. Thus, neither small metastases nor viable aerobic regions of the primary tumour can be targeted with such spores.

To overcome this limitation, researchers focus on facultative anaerobic bacteria for BMTT. Accordingly, *Salmonella* Typhimurium can target both aerobic and anaerobic tumour regions (Ruby *et al*., 2012). How *Salmonella* is able to invade solid tumours is still under debate, but a reasonable explanation for the mechanism is already specified: a cytokine storm is elicited upon intravenous or intraperitoneal application of the bacteria that is usually dominated by TNF‐α. This cytokine opens the pathological blood vessels of the tumour and results in a haemorrhage and consequently in the formation of a large necrotic area. Due to the high blood influx, the bacteria are flushed into the tumour. They then thrive in the necrotic hypoxic/anoxic environment where they are protected from the immune system (Westphal *et al*., 2008; Leschner *et al*., 2009). However, it remains still unclear whether tumour colonization is essential for therapeutic efficacy or whether the therapeutic power of the bacteria is due to an adjuvant effect exerted by the bacteria exclusively in immune inductive sites.

While the ability of *Salmonella* to grow in the presence of oxygen might be beneficial for tumour therapy, it enables *Salmonella* to also target healthy tissues like spleen and liver. Thus, in principle, *Salmonella* is not as tumour specific as *Clostridia*. Therefore, *Salmonella* needs to be attenuated in a way that it can only survive and inflict damage inside the immune‐privileged site of the tumour. Unfortunately, attenuating *Salmonella* for cancer therapy is not a straightforward process. Many studies have shown that inactivating essential genes easily results in overattenuated therapeutic strains (Schmitt *et al*., 1994; Frahm *et al*., 2015). Although safe in various animal models, the antitumour properties might not be preserved. Thus, the major challenge of using *Salmonella* in BMTT is to find an appropriate balance of safety and therapeutic efficacy.

In the past decades, several strategies have been followed to find such a balance. First approaches aimed to passage *Salmonella* through cancerous tissue either *in vitro* or *in vivo* to improve tumour‐targeting by selection. This strategy resulted in the prominent *Salmonella* vector strains VNP20009 and A1‐R (Low *et al*., 2004; Zhao *et al*., 2006; Zhang *et al*., 2017). VNP20009 was evaluated in clinical trials. However, it turned out that VNP20009 was overattenuated due to the uncontrolled introduction of multiple deletions during the selection process (Toso *et al*., 2002; Broadway *et al*., 2015). This might explain the ineffectiveness of this strain in human patients. The *Salmonella* variant A1‐R, on the other hand, appears to be highly potent. This strain is deficient for the amino acids arginine and leucine and has shown great promise in many different types of murine tumours and patient‐derived orthotopic xenografts (Hoffman, 2016; Zhang *et al*., 2017). It will be interesting to follow this particular strain and its performance in clinical trials with human cancer patients.

In addition to random selection, another promising strategy is to design *Salmonella* strains by targeted gene deletion, which avoids unexpected modifications. Prominent candidates by this approach include the magic spot mutant of *Salmonella* (*ΔppGpp*) that was enhanced by expressing a secreted flagellin (Yun *et al*., 2012; Zheng *et al*., 2017), and SF200 which comprises several mutations affecting LPS and flagella synthesis, as well as containing metabolic modifications (unpublished data). Both strains have been shown to be tumour specific and exhibit high efficacy in various murine tumour models. Strain SF200 is interesting in particular because it was constructed in a modular way. Thereby, the bacteria are not only attenuated but at the same time, the immune stimulatory capacities were increased by targeted modifications (Felgner *et al*., 2016b; Kocijancic *et al*., 2017). As consequence, SF200 is the first therapeutic strain able to overcome pre‐existing anti‐*Salmonella* immunity and retains its therapeutic efficacy under such conditions. It may counter one of the major obstacles for BMTT and could be a promising candidate for clinical trials.

In summary, many of these studies have shown that bacteria can target and retard the growth of several tumours, and even clear these neoplasia in some cases. However, many tumours still withstand the therapy after an initial phase of response. Thus, researchers shift to either (i) use the bacteria as a vector system that shuttles therapeutic substances, e.g. rIL‐2, sh‐RNAs, CPG2, cytosine deaminase, as well as toxins like α‐haemolysin into tumours (Nemunaitis *et al*., 2003; Friedlos *et al*., 2008; Nguyen *et al*., 2010; Blache *et al*., 2012; St. Jean *et al*., 2014); or (ii) adjuvant therapy, where the bacteria are applied together with checkpoint inhibitors like α‐PD‐1 and α‐CTLA‐4 or together with adoptively transferred anticancer T cells (Binder *et al*., 2013, 2016; Hiroshima *et al*., 2014, 2015). To avoid safety concerns, another approach is to replace the bacterial vector by nanoparticles that shuttle bacterial components into tumours (Felgner *et al*., 2016c; Mercado‐Lubo *et al*., 2016). However, the specificity and side‐effects of such therapies remain unclear and are still subject to investigations. Furthermore, bacteria can be also employed for cancer diagnostics as demonstrated recently (Yu *et al*., 2004; Danino *et al*., 2015). This possibility has been little explored thus far but the great potential became apparent.

Taken together, the unique intrinsic properties of bacteria to specifically colonize cancerous tissue and to elicit an antitumour response combined with their potential as a targeted delivery vector system provide a solid platform for cancer therapy with an extremely high potency. It represents a perfect example how the delivery and quality of therapeutics can be improved. From the first attempts to revive Coley's strategy (Pawelek *et al*., 1997) until today, dramatic progress has been made in understanding the process but also in enhancing the bacteria by genetic modifications. This progress will continue. Thus, BMTT will grow into a versatile alternative to conventional therapies that is not restricted to a particular type of tumour. In fact, microbial therapy may become one of the most specific therapies for cancer treatment besides its potential in cancer prevention and biotechnological diagnostic. Thus, BMTT has the potential to help to release mankind from the curse of cancer.

## Conflict of interest

The authors declare that there is no conflict of interest regarding the publication of this paper.
